# Hypoxia and hypoxia-mimetics attenuate the inflammatory response during murine endotoxemia

**DOI:** 10.1186/2197-425X-3-S1-A421

**Published:** 2015-10-01

**Authors:** D Kiers, R Groeneveld, JG van der Hoeven, GJ Scheffer, P Pickkers, M Kox

**Affiliations:** 1 Department of Intensive Care Medicine, Radboud University Medical Center, Nijmegen, Netherlands; 2 Department of Anesthesiology, Radboud University Medical Center, Nijmegen, Netherlands

## Introduction

Hypoxia has been shown to exert immunomodulatory effects^1^. As oxygenation is daily practice in critical care, and the majority of critically ill patients suffer from inflammatory-related conditions, *permissive hypoxia* might be a novel therapeutic strategy. In addition, there are pharmacologic hypoxia-mimetics available that can replicate the hypoxia-effects without the potential drawbacks of systemic hypoxia. The hypoxic immunomodulatory effects are thought to be mediated through a group of transcription factors called hypoxia-inducible factors (HIFs)^2^. However, *in vitro* studies have demonstrated that, depending on the cell-type, these effects can be both pro- and anti-inflammatory. The net effects of hypoxia during systemic inflammation *in vivo* are therefore unknown.

## Objectives

To determine the immunomodulatory effects of various degrees of hypoxia and hypoxia mimetics during systemic inflammation in mice.

## Methods

BALB/c mice (n = 8 per group) were placed in an air-tight cage with variable degrees of oxygen (normal (21%), 12%, 9%, and 6%), or were injected with the hypoxia-mimetic cobalt chloride (CoCl_2,_ 30mg/kg i.p.). After 1 hour, LPS (5 mg/kg *E. Coli* endotoxin, serotype 0111:B4) or placebo (NaCl 0.9%) was administered i.p. Ninety minutes after LPS/placebo administration, rectal temperature was measured and animals were sacrificed. Blood plasma was analyzed for cytokine concentrations. Furthermore, mRNA expression of interleukin (IL)-10 and the HIF-1α target gene vascular endothelial growth factor (VEGF) were determined in spleen samples.

## Results

**Figure 1:**
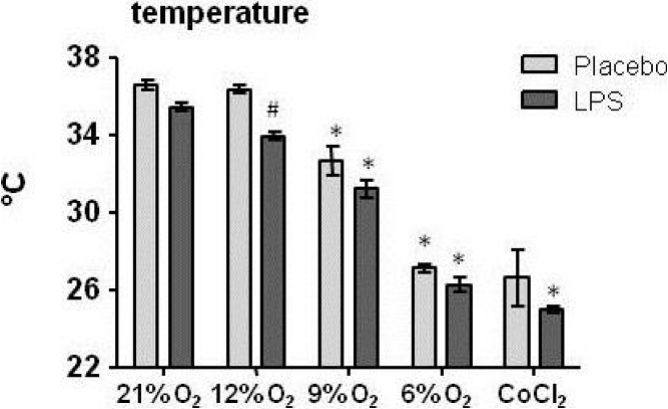
Rectal temperature in degrees Celsius (°C). Data are shown as mean ± SEM. Stastistical analysis was performed using two-way analysis of variance with Bonferonni post-hoc tests. * p < 0.05 compared with normoxia (21% with same LPS/placebo) # p < 0.05 compared with placebo (same % oxygen or CoCl2).

**Figure 2:**
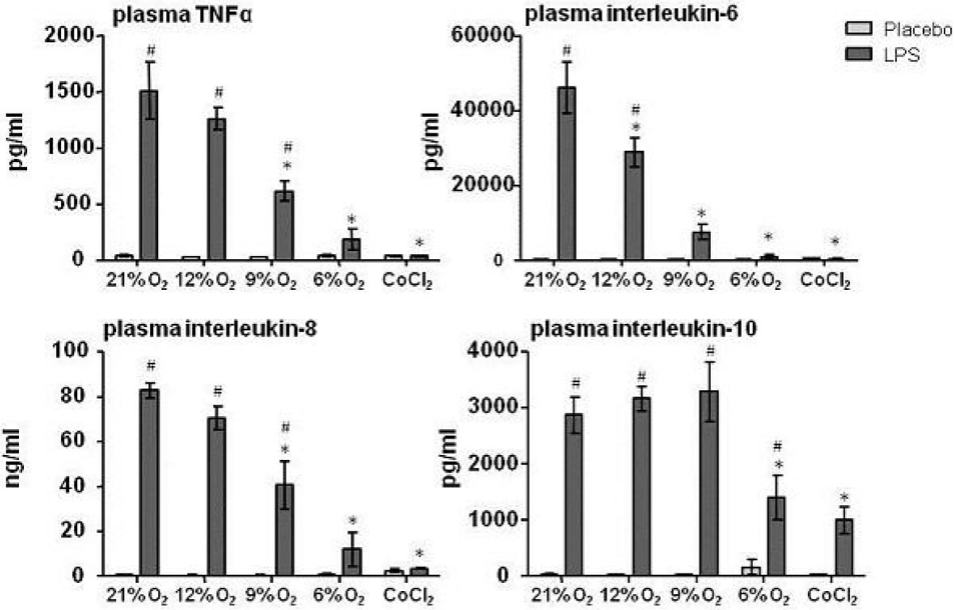
Plasma cytokines (Tumor Necrosis Factor (TNF)α, Interleukin(IL)-6, IL-8 and IL-10). Data are shown as mean ± SEM. Statistical analysis was performed using two-way analysis of variance with Bonferonni post-hoc tests. * p < 0.05 compared with normoxia (21% with same LPS/placebo). # p < 0.05 compared with placebo (same % oxygen of CoCl2)

**Figure 3:**
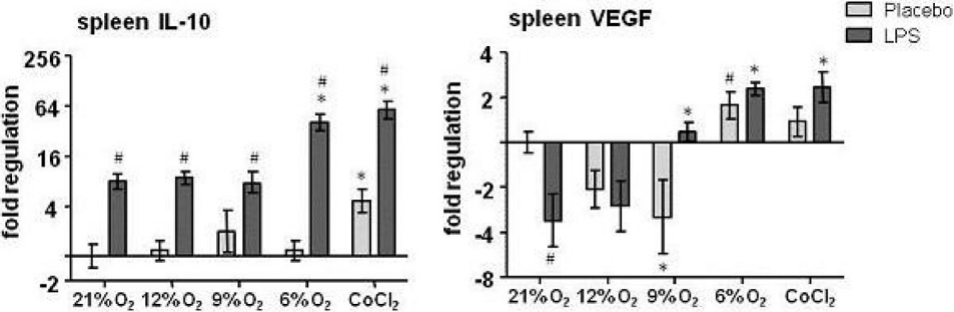
Splenic mRNA expression of Interleukin 10 (IL-10) and vascular endothelial growth factor (VEGF). Data are shown as mean ± SEM. Statistical analysis was performed using two-way analysis of variance with Bonferonni post-hoc tests. * p < 0.05 compared with normoxia (21% with same LPS/placebo). # p < 0.05 compared with placebo (same % oxygen of CoCl2)

As expected, LPS administration resulted in hypothermia. Hypoxia and CoCl_2_ also lowered body temperature, in a dose-dependent fashion (Figure 1). Hypoxia itself did not result in elevated cytokine levels in plasma. Endotoxemia resulted in increased levels of circulating pro-inflammatory cytokines Tumor Necrosis Factor (TNF)-α, IL-6, IL-8, as well as anti-inflammatory IL-10 (Figure 2). Hypoxia and CoCl_2_ attenuated the endotoxin-induced pro-inflammatory cytokine response in a dose-dependent manner, while IL-10 protein levels were relatively unaffected. Furthermore, hypoxia resulted in a dose-dependent upregulation of splenic VEGF and IL-10 mRNA expression (Figure 3).

## Conclusions

Hypoxia results in hypothermia and attenuation of the systemic pro-inflammatory response in a dose-dependent fashion, while preserving or enhancing the anti-inflammatory response. Administration of the hypoxia-mimetic CoCl_2_ results in a similar immunological phenotype. Our results suggest that permissive hypoxia is a novel non-pharmacological anti-inflammatory therapeutic strategy.
